# Effect of Gum Arabic powder on the mechanical properties of denture base acrylic

**DOI:** 10.12669/pjms.39.1.6937

**Published:** 2023

**Authors:** Omar Alsadon, Abdulaziz Abdullah Alkhureif, Aftab Ahmed Khan

**Affiliations:** 1 Omar Alsadon, PhD, MPhill, BDentTech, Assistant Professor, Dental Health Department, College of Applied Medical Sciences, King Saud University, Riyadh, Saudi Arabia; 2 Abdul Aziz Abdullah Alkhureif, PhD, MPhill, BDentTech, Professor, Dental Health Department, College of Applied Medical Sciences, King Saud University, Riyadh, Saudi Arabia; 3 Aftab Ahmed Khan, PhD, MSc, M.Bioeth, B.D.S. Researcher, Dental Health Department, College of Applied Medical Sciences, King Saud University, Riyadh, Saudi Arabia

**Keywords:** Gum Arabic, Denture base acrylic, poly methyl methacrylate (PMMA), Micro hardness, Fracture toughness

## Abstract

**Objective:**

This study aimed to improve the mechanical properties of denture base material using various concentrations of natural biopolymer, i.e., Gum Arabic (GA).

**Methods:**

This experimental study was conducted at the Dental Health Department of the College of Applied Medical Sciences, King Saud University, Riyadh, Saudi Arabia from May 2022 to July 2022. After obtaining exemption from the institutional review board, the powdered GA was added in ratios of weight 5, 10, and 20% to PMMA heat-cured acrylic powder to produce bar-shaped samples (65 × 10 × 30 mm^3^ in dimensions). While the control group was prepared as such. Micro hardness (n=10/group) and fracture toughness (n=10/group) were evaluated. One-way analysis of variance method was used to statistically analyze the results (p<0.05) using SPSS version 23.

**Results:**

Significant differences were observed for micro hardness (p<0.001) and fracture toughness (p=0.007) between the means of the different study groups. The control group exhibited the highest micro hardness (22.5±0.6 VHN) and fracture toughness (1.25±0.11 MPa.m^1/2^) value among the study groups. While 20 wt. % GA and 10 wt. % GA groups showed the lowest micro hardness and fracture toughness values, respectively.

**Conclusions:**

GA powder might not be an appropriate reinforcing material for denture base or the higher filler loading of GA in denture base acrylic might be detrimental to the mechanical properties.

## INTRODUCTION

Even though implant prostheses are widely used to replace missing teeth, however removable partial/full dentures are still considered viable and popular tooth replacement options.^1^,^2^ Due to their good operating features such as ease of repair, cost-effectiveness, and stability in the oral environment, polymethyl methacrylate (PMMA) has been the material of choice.^3^,^4^ However, PMMA-based denture bases are vulnerable to fracture.^5^ It is a common clinical problem associated with dentures, and is still an unsolved problem.

The fatigue of denture material is the primary cause of fractures.^6^ In the fatigue mechanism, flexural loads over time cause and create microscopic cracks that propagate through the denture base.^7^ Micro-fissures can occur in the denture material due to continual pressure from the masticatory load, resulting in midline fractures and flexural strain.^8^ Mastication is a recurrent force that causes widespread cracking, causing the denture’s base to weaken and eventually fracture.^9^ When evaluating the duration of use and clinical durability of a denture, flexural fatigue resistance and fracture toughness are the most critical mechanical attribute of the denture base material.^5^

Approaches have been employed to enhance the mechanical properties of a denture base material including chemical composition modifications, different polymerization techniques, and/or the addition of reinforcement materials including bioactive glasses.^10^ However, the techniques failed because of one reason or another. Incorporation of metal fillers is also found to be difficult and technique sensitive, also their bonds with the resin matrix are weak.^11^

Gum Arabic is a natural polymer with antimicrobial activity. It is also a non-toxic natural excipient used to deliver the bioactive formula in the sustained release of drugs.^12^ The addition of rubbery material slows down the fatigue process and crack propagation in denture base acrylic.^13^ Moreover, this rubbery material has carboxyl groups which have the affinity to bind with the chemical groups of the PMMA.^14^ Use of Gum Arabic can be a viable alternative to existing synthetic fibers. To our best knowledge, there is no research available on the applicability of these natural polymeric fibers for enhancing the mechanical properties of a denture base. Therefore, this research focused to evaluate the effect of the incorporation of Gum Arabic in PMMA denture acrylic with different wt. %. It was hypothesized that Gum Arabic would increase the mechanical properties of denture base material.

## METHODS

This experimental study was performed over three months at the Dental Health Department, College of Applied Medical Sciences, King Saud University Saudi Arabia from May 2022 to July 2022. Institutional Review Board exempted the research because no human or animal subjects were involved. In this laboratory study, acacia gum (i.e. Gum Arabic) was obtained and ground to 80-150 μm in size using a ball-milling device (Firtsch Pulverisette 5, Duisburg, Germany). The powdered acacia Gum was added in ratios of weight 5, 10, and 20% to PMMA heat-cured acrylic powder, i.e., Interacryl hot (Interdent, Opekarniska, Slovenia) to produce a powder mixture of three experimental denture bases (i.e., 5 wt.% GA, 10 wt.% GA & 20 wt.% GA). While the control group was prepared with 0 wt. % GA content. The experimental mixtures were dispersed vigorously for five minutes using a vacuum mixer.

The powder/monomer ratio of 2.3:1.0 by weight was mixed manually using a rubber bowl and a stainless steel spatula until the mixture solution acquired a dough stage. Next, the mixture was placed in a gypsum mold present in a dental flask (dimensions of 65 × 10 × 3 mm^3^). A load of 100 N was then administered to the flask to remove the excessive acrylic material after which the polymerization occurred in a water bath (eight hours at 73ºC) and then increase the temperature to 100°C for one hour.^15^

The polymerized rectangular acrylic blocks were then removed from the flasks and cut (dimensions of 65 × 10 × 3 mm^3^, n = 10) with an electric saw device (Isomet 5000; Buehler Ltd, Lake Bluff, IL, USA) at 1600 rpm. The specimens were ground with 320-grit silicon carbide paper to obtain polished surfaces. The cut specimens were stored in a desiccator for 24 hour before performing any mechanical test.

### Micro hardness test:

The average micro hardness of the GA incorporated PMMA-based composite samples was calculated by NOVA 130 micro hardness tester (Innovates, Netherlands) at a load of 300g and dwell time of 15s. Each specimen had its surface indented three times. The specimen micro hardness was calculated as the average of the three indentations.

### Fracture toughness test:

The specimens were prepared following ISO 13586:2000, and the single edge notch test was performed to assess the fracture toughness. The mold’s internal dimensions for the specimens (n=10/group) were predetermined to be as follows: a notch length (a) of four mm, a support span length (s) of 64 mm, and the overall length was 80 mm. While width (w) was 20 mm and thickness (t) were four mm. Peak load (P) to fracture measurements were made during fracture toughness tests using a universal testing device (Model 4301; Instron, Canton, MA, USA). KIC = (3PSa^1/2^ y)/ (2tw^2^) While the geometrical correction factor (y) = 1.93–3.07 (a/w) + 14.53 (a/w) ^2^ – 25.11 (a/w) ^3^ + 25.8 (a/w).^4^

### Statistical analysis:

The study groups were evaluated with both descriptive and inferential statistics. For inferential statistics, one-way analysis of variance (ANOVA) followed by post hoc Tukey’s HSD multiple comparison tests (p=0.05) were employed. The SPSS 23.0 statistical program (IBM, Armonk, NY, USA) was used for the statistical analyses.

## RESULTS

The outcome of one-way ANOVA is presented in Table-I. The outcome suggests that there was a statistically significant difference between the means of the different materials groups (P=0.000) on the dependent variable, i.e., micro hardness.

**Table-I:** One-way analysis of variance for dependent variable micro hardness.

	Sum of Squares	df	Mean Square	F	Sig.
Between Groups	46.181	3	15.394	30.007	.000
Within Groups	8.208	16	.513		

Total	54.389	19			

Table-II: Presents the outcome of one-way ANOVA. The outcome suggests that there was a statistically significant difference between the means of the different materials groups (P<0.05) on the dependent variable, i.e., fracture toughness.

**Table-II:** One-way analysis of variance for dependent variable fracture toughness.

	Sum of Squares	df	Mean Square	F	Sig.
Between Groups	.198	3	.066	5.742	.007
Within Groups	.184	16	.011		

Total	.382	19			

**Fig.1:**
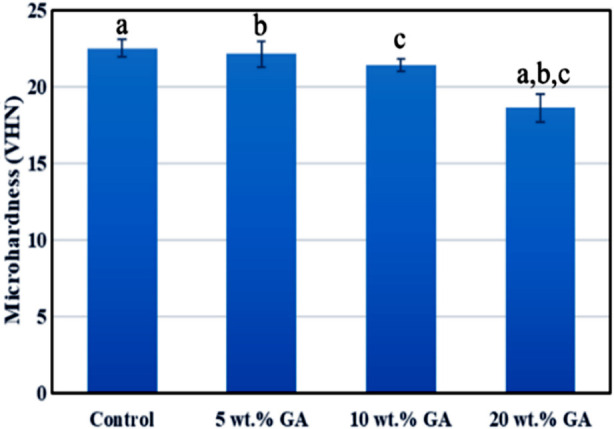
Bar chart of micro hardness means values of the control and experimental groups same lowercase letters show statistical difference between the study groups.

Fig.1: Presents the mean micro hardness values of the study groups. The highest micro hardness was demonstrated by the control group (22.5±0.6 VHN). While 20 wt. % GA group showed the lowest micro hardness value (18.7±0.9 VHN). The *post hoc test* revealed the statistical difference between the groups (p<0.05). Fig.1

**Fig.2:**
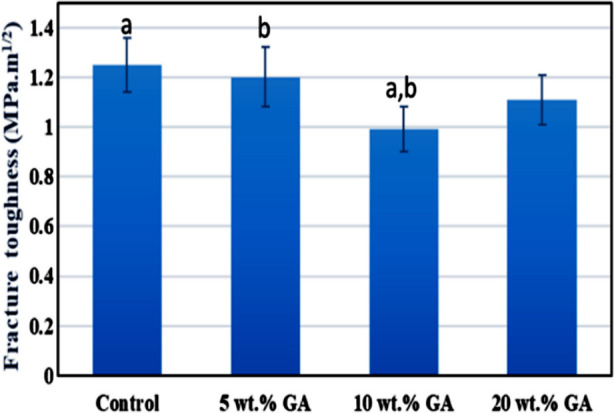
Bar chart of fracture toughness means values of the control and experimental groups same lowercase letters show statistical difference between the study groups.

`Fig.2: Represents the mean fracture toughness values (in MPa.m^1/2^) of the study groups. The highest was observed in the control group (1.25±0.11 MPa.m^1/2^) while 10 wt. % GA groups demonstrated the lowest fracture toughness value (0.99±.09 MPa.m^1/2^). The post hoc test revealed statistical differences between the study groups (p<0.05). The details are presented in (Fig.2).

## DISCUSSION

The micro hardness and fracture toughness of the denture base acrylic tended to reduce with the incorporation of GA micro-sized powder in this study. Hence, the null hypothesis is rejected: incorporation of GA in denture base acrylic had a deleterious effect on the mechanical properties of dental base acrylic. Although the white color of GA powder did not compromise the aesthetic appearance of the dental base acrylic. However, the decreased micro hardness and fracture toughness values among the experimental groups might hint toward several factors such as weak bonding between GA particles and acrylic resin, inhomogeneous dispersion, or agglomeration of GA powder in acrylic resin, and porosity in the GA-containing samples. The GA particles are hydrophilic, they tend to agglomerate with each other when dispersed in PMMA denture acrylic.^16^

Surface hardness determines the suitable material to be selected since the denture base is subjected to scratching and abrasive pressures during clinical use or mechanical cleaning.^17^,^18^ in this study, the effects on micro hardness were correlated with the amount of GA powder incorporated in acrylic resin. The gradual decrease in micro hardness with the increasing concentration of GA powder might be attributed to the accumulation of micron-sized GA into the acrylic resin, especially on the surface. GA is a natural polysaccharide with rubbery properties.^19^ Due to its rubbery nature, the addition of GA powder weakened the surface micro hardness of acrylic resin. Additionally, the decreased micro hardness in experimental groups could be attributed to the higher wt. % of GA in denture base acrylic.

Because of the hydrophilic nature, organic reinforcing GA powder exhibit high surface energy. However, due to the disparity in surface energy, the hydrophobic resin acrylic polymer might not wet or interact with the GA particles.^20^ It is usually advised to modify the reinforcing agent surface to improve surface wetting and bonding between the reinforcing agent and acrylic resin.^21^ Hence, in this study the reinforcing GA powder was treated with three-Methacryloxypropyltrimethoxysilane (MPS) saline. MPS saline is the most frequently used saline in dental materials^22^, and is considered to strengthen the bonding between the acrylic resin and the filler.^23^ However, in our study, MPS did not improve the mechanical properties or the loading of the reinforcing agent was not optimal.

Previous studies have also advocated low filler loading for enhanced fracture toughness.^24^,^25^ It is assumed that there is a threshold limit of filler incorporation. Beyond that limit, detrimental effects on the mechanical properties of the denture base acrylic might be seen. Al-Bakri et al. had advocated 10 wt.% as the threshold limit.^1^ Due to fatigue and impact, dentures frequently fracture in the middle; therefore, toughness is essential to avoid denture fractures.

We evaluated the three-point fracture toughness test; however, this test has limitations because the applied force is only in one direction. While in the mouth multidirectional and grinding forces are active simultaneously.^6^ In our study, the experimental groups showed reduced fracture toughness. The findings suggest that micron-sized GA powder did not demonstrate to stop indiscriminate crack propagation. Moreover, the incorporated powder might produce porosity in the acrylic resin. The formation of voids at the interfacial area between GA powder particles and acrylic resin caused the lower fracture toughness in experimental groups. Because of the uniqueness and unavailability of any previous published data, this study cannot be compared with any laboratory or clinical study.

### Limitations of the study:

In laboratory investigations, there always remain some limitations that need further elaboration. In this study, the other important mechanical properties such as tensile, impact strength, etc. were not evaluated. In addition, the concentration of the reinforcing agent used was high. In the future, it is recommended to evaluate the effect of GA powder using a lower concentration in acrylic resin. It would also be interesting to use nano-sized instead of micron-sized GA powder to see the effect on reinforcing the denture base.

## CONCLUSION

This study demonstrated that the incorporation of GA micron-sized powder into denture acrylic resin is ineffective and reduced the micro hardness and fracture toughness of the denture base acrylic. Micro hardness and fracture toughness decreased with the increasing weight % of GA powder in denture base acrylic. However, there needs research on uniform distribution of GA filler in denture base acrylic and the impact of reduced filler loading on mechanical properties.

### Authors’ Contribution:

**OA** did data collection, data interpretation, and manuscript writing and is responsible for the accuracy and integrity of the research.

**AAA** conceived, supervised the study project and approval of manuscript writing.

**AAK** did statistical analysis, tabulation of the data, revision, formatting and editing of the final draft.
